# Supplementary material for: SARS-CoV-2 Nonstructural Proteins 1 and 13 Suppress Caspase-1 and the NLRP3 Inflammasome Activation
Source: Microorganisms. 2021 Feb 26;9(3):494. doi: 10.3390/microorganisms9030494 (PMC7996899; doi:10.3390/microorganisms9030494)
Supplement: Supplementary file 1 [file microorganisms-09-00494-s001.pdf]

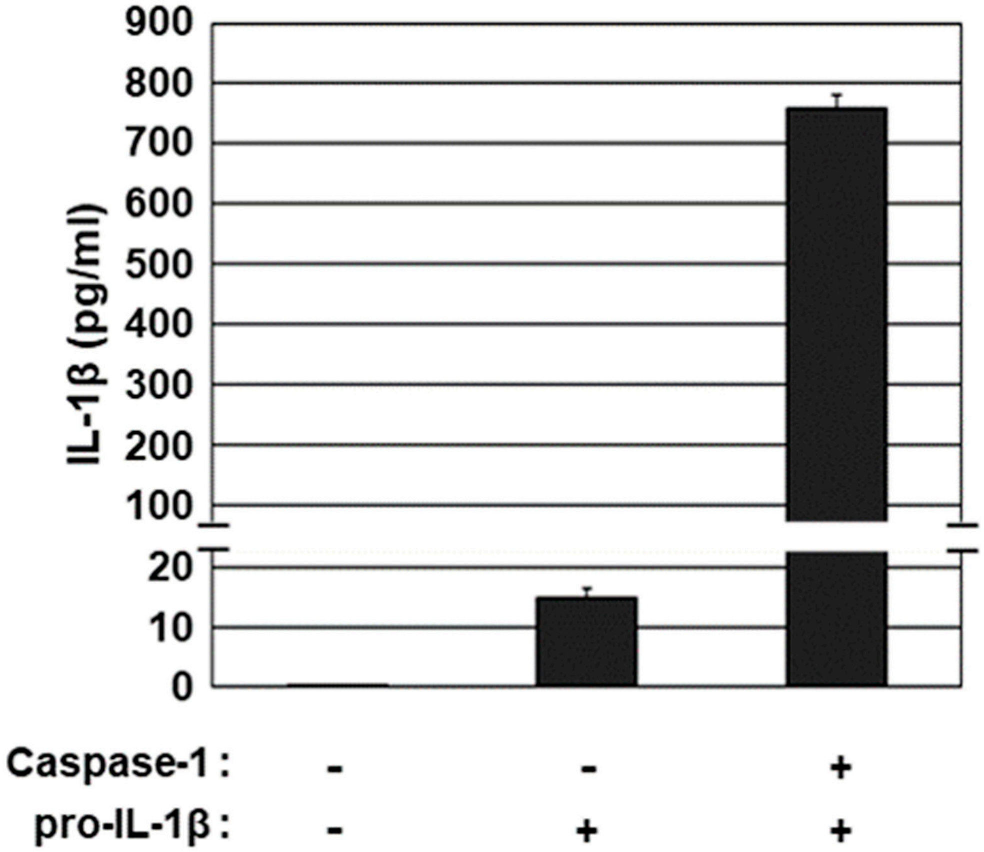

Figure S1. Caspase-1-mediated IL-1β secretion. HEK293T cells plated at 8.0 x 10^5 cells/well in 6 well plates were transfected with a vector expressing pro-IL-1β with or without a vector expressing caspase-1. After 24 h, the secretion of mature IL-1β into culture supernatants was measured by ELISA. Data shown are representative of three independent experiments.

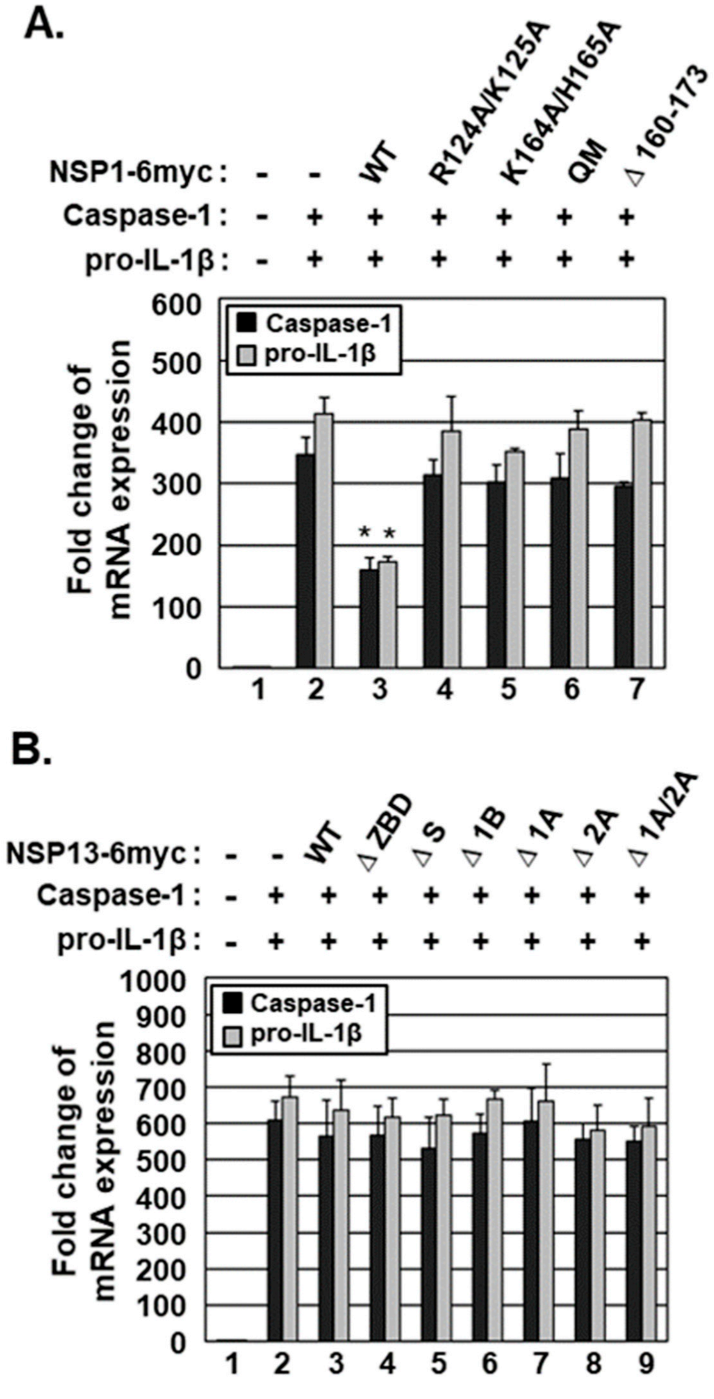

Figure S2. Effects of NSP1 and NSP13 on caspase-1 and IL-1β transcripts. HEK 293T cells were co-transfected with vectors expressing caspase-1 and pro-IL-1β plus either pEF1α-DEST

or pEF1α-DEST expressing (A) c-Myc-tagged NSP1 WT or NSP1 containing the mutations R124A/K125A, K164A/H165A, QM and Δ 160-173 or (B) c-Myc-tagged NSP13 WT or mutants carrying deletions of the ZBD (ΔZBD), the stalk domain (ΔS), the 1B domain (Δ1B), the two RecA-like domains, 1A (Δ1A) and 2A (Δ2A) and both 1A and 2A (Δ1A/2A). After 24 h, the relative levels of caspase-1 and IL-1β mRNAs were measured by qRT-PCR. Caspase-1 and IL-1β mRNA levels in control cells were set as 1. $*P < 0.001$ by unpaired two-tailed Student's *t*-tests. Data shown are representative of three independent experiments.

Table S1. List of primers used to generate NSP1 and 13 mutants

Primer	Sequence
NSP1 R124A/K125A F	CTTACAGAAAGGTGCTGCTGGCTGCGAACGGAAACAA
NSP1 R124A/K125A R	CGGCACCCTTGTTTCCGTTCGCAGCCAGCAGCACCTTTCTGTAAG
NSP1 K164A/H165A F	CAGGAAAACTGGAACACCGCGGCCAGCTCCGGAGTGACCAG
NSP1 K164A/H165A R	CTGGTCACTCCGGAGCTGGCCGCGGTGTTCCAGTTTTCCTG
NSP1 Δ160-173 F	ATGCGTGAACTGAACGGT
NSP1 Δ160-173 R	TTCCTGGAAGTCCTCGTAA
NSP13 ΔZBD F	CATGCCAACTTTTTTGTACAA
NSP13 ΔZBD R	TCCGACAACGTGACCGA
NSP13 ΔS F	GCCCACGCAGGTGTTCTT
NSP13 ΔS R	GGTATCGCTACCGTGCGT
NSP13 Δ1B F	GTAAGACAGCTTGAAGGT
NSP13 Δ1B R	GAGTTCAGCTCCAACGTG
NSP13 Δ1A F	GTCGGAGATGTTCAGGGT
NSP13 Δ1A R	CGCCGCTGCCCTGCTGAA
NSP13 Δ2A F	GCAGGTACCCAGGAACAT
NSP13 Δ2A R	TACCCAACTTTCTTGTACA
